# Neuroprotectin D1 Attenuates Laser-induced Choroidal Neovascularization in Mouse

**Published:** 2010-03-02

**Authors:** Kristopher G. Sheets, Yongdong Zhou, Monica K. Ertel, Eric J. Knott, Cornelius E. Regan, Jasmine R. Elison, William C. Gordon, Per Gjorstrup, Nicolas G. Bazan

**Affiliations:** 1 Department of Ophthalmology and the Neuroscience Center of Excellence, Louisiana State University Health Sciences Center, New Orleans, LA; 2 Resolvyx Pharmaceuticals, Inc., Bedford, MA

## Abstract

**Purpose:**

To examine the effects of neuroprotectin D1 (NPD1), a stereospecific derivative of docosahexaenoic acid, on choroidal neovascularization (CNV) in a laser-induced mouse model. Specifically, this was assessed by clinically grading laser-induced lesions, measuring leakage area, and volumetrically quantifying vascular endothelial cell proliferation.

**Methods:**

C57Bl/6 mice were treated with vehicle control or NPD1, and choroidal neovascularization was induced by laser rupture of Bruch's membrane; treatment was administered throughout the first week of recovery. One and two weeks after CNV induction, fundus fluorescein angiography was performed. Angiograms were clinically graded to assess leakage severity, while leakage area was measured by image analysis of angiograms. Proliferation of vascular endothelial cells was evaluated volumetrically by three-dimensional laser confocal immunofluorescent microscopy of cytoskeletal, nuclear, and endothelial cell markers.

**Results:**

At seven days after CNV induction, NPD1-treated mice had 60% fewer clinically relevant lesions than controls, dropping to 80% fewer by 14 days. NPD1 mice exhibited 25% smaller leakage area than controls at 7 days and 44% smaller area at 14 days. Volumetric immunofluorescence revealed 46% less vascular endothelial cell volume in 7-day NPD1-treated mice than in 7-day controls, and by 14 days NPD1 treatment was 68% lower than controls. Furthermore, comparison of 7- and 14-day volumes of NPD1-treated mice revealed a 50% reduction at 14 days.

**Conclusions:**

NPD1 significantly inhibits choroidal neovascularization. There are at least two possible mechanisms that could explain the neuroprotective action of NPD1. Ultimately, nuclear factor-κB could be inhibited with a reduction in cyclooxygenase-2 (COX-2) to reduce vascular endothelial growth factor (VEGF) expression, and/or activation of the resolution phase of the inflammatory response/survival pathways could be upregulated. Moreover, NPD1 continues to be effective after treatment is concluded, suggesting sustained protection and highlighting the potential applicability of this lipid mediator in preventing or ameliorating endothelial cell growth in pathoangiogenesis.

## Introduction

Neovascularization at or near the retina/vitreous interface is characteristic of retinopathy of prematurity and diabetic retinopathy, while new vessel growth from the choroid into the retina occurs in age-related macular degeneration (AMD). Choriocapillaris endothelial cell proliferation is stimulated when Bruch’s membrane is damaged. In hypoxic/damaged regions, endothelial cells divide, align, tubularize, and lay down new basement membrane to form functioning vessels. During this process, newly formed capillaries leak and displace surrounding cells [1]. Ramification of these new vessels through the retinal pigment epithelial (RPE) cell layer into the subretinal space may lead to retinal detachment and hemorrhaging, inducing photoreceptor cell death and causing loss of the central visual field. This choroidal neovascularization (CNV) is characteristic of wet or exudative AMD. While the physiologic, cellular, and biochemical events leading to AMD are not well defined, inflammation has been shown to be an early event [2].

The pro-inflammatory cytokines tumor necrosis factor α (TNF-α) and interleukin (IL)-1β increase the permeability (i.e., vascular leakage) of the pericyte/endothelial cell unit through a phospholipase A_2_ (PLA_2_)-dependent mechanism [3]. Activation of the pro-inflammatory transcription factor nuclear factor κB (NF-κB) is a common downstream component of both TNF-α and IL-1β pathways. Our laboratory has shown in retinal choroid cells that IL-1β-induced NF-κB activity ultimately leads to upregulated vascular endothelial growth factor (VEGF) expression [4]. VEGF controls both physiologic and pathological angiogenesis [5], and increased expression of VEGF is associated with AMD pathology [6]. VEGF-neutralizing agents, primarily as an antibody-based approach, have been successful in reducing angiogenesis in animal models [7], and pharmacological intervention, based on these principles is now being used in the treatment of AMD [8]. Thus, one approach for managing AMD is inhibition of the signaling that triggers vascularization [9], although this is a secondary event resulting from primary insults. However, a second possibility for intervention involves the upstream regulation of VEGF-triggering events, including upregulation of protective signaling that can suppress/attenuate the initial induction of the pro-angiogenic pathway.

Lipid mediators have key functions in the cell-to-cell signaling of the retina and in other parts of the nervous system, and these mediators display both pro- and anti-inflammatory bioactivity. For example, the ω-6 arachidonic acid derivatives prostaglandin E_2_ (PGE_2_) and leukotriene B_4_ (LTB_4_) elicit pro-inflammatory responses by increasing the production of IL-6. Conversely, the ω-3 polyunsaturated fatty acid (PUFA) docosahexaenoic acid (DHA) is anti-inflammatory [10]; decreases cytokines, adhesion molecules, and reactive oxygen species; and regulates cyclooxygenase-2 (COX-2) activity [11]. PUFAs, especially DHA and arachidonic acid, are abundant in retina where they become concentrated in photoreceptor and RPE cells [12] and the retinal vasculature [13]. If released from the membranes of these cells by PLA_2_ under conditions of stress, PUFAs become precursors for a variety of mediators involved in the resolution of inflammation. These include prostaglandins, lipoxins, resolvins [11], and the docosanoid neuroprotectin D1 (NPD1; 10R, 17S-dihydroxy-docosa-4Z, 7Z, 11E, 13E, 15Z, 19Z-hexaenoic acid) [14-16]. Connor et al. [17] have shown that NPD1, resolvin D1, and resolvin E1 (all derivatives of ω-3 PUFAs) suppress angiogenesis, and Szymczak et al. [18] demonstrated that this particular process is mediated by upregulation of COXs. Because the ω-3 PUFAs inhibit major pro-angiogenic processes in endothelial cells [18] but do not affect VEGF [17], ω-3 PUFA protection may involve a VEGF-independent mechanism.

DHA is enriched and selectively retained in photoreceptor cells where it is incorporated into synapses and outer segment disk membranes [19,20]. Reduced retinal DHA leads to rod-based visual dysfunction [21], while an abnormally low amount of brain DHA promotes learning deficits [22]. In addition, DHA is protective of neuronal and RPE cells undergoing oxidative stress [15,16,23-25] and of brain neurons during transient, focal, cerebral ischemia [26]. NPD1 is a DHA-derived stereoselective mediator that rescues RPE cells and bestows neuroprotection in a model of stroke [26,27]. Moreover, NPD1 protects against neovascularization in a mouse model of oxygen-induced retinopathy [17]. Therefore, NPD1 may prove effective in depressing endothelial cell growth from the choriocapillaris into the photoreceptor layer in a laser-induced mouse model of CNV. However, while vascular proliferation is a hallmark of several degenerative diseases in retina, laser-induced choroidal neovascularization is an experimental approach to produce an injury rather than a model for AMD.

In the present study we evaluated the effect of NPD1 on the growth of endothelial cells from the choriocapillaris into the subretinal space and the subsequent leakage in the retina of these new pathogenically formed vessels.

## Methods

All animal experiments conformed to the Association for Research in Vision and Ophthalmology (ARVO) statement for the Use of Animals in Ophthalmic and Vision Research and were approved by the Institutional Animal Care and Use Committee for the Louisiana State University Health Sciences Center (LSUHSC). Male C57Bl/6 mice (25–30 g) were obtained from Charles River Laboratories (Wilmington, MA) and maintained in the LSUHSC animal colony on a 12 h:12 h light–dark cycle (0600 h on; 1800 h off) at an average illumination of 20 lux (bedding level in cage center on ventilation rack). Animals were fed normal mouse chow and supplied with water ad libitum.

Prior to laser treatment and during subsequent times when retinal imaging occurred, 70 mice were anesthetized with intraperitoneal (i.p.) injections of 100 mg/kg ketamine and 10 mg/kg xylazine (Vedco Inc., St. Joseph, MO). Pupils were dilated with 1% Tropicamide Ophthalmic Solution (Tropicacyl^®^; Akorn, Inc., Buffalo Grove, IL), and contact lenses (Veterinary Specialty Products, Whitechurch, UK) were used to improve image quality and prevent corneal dessication. NPD1 (1 mg/ml in 97.5% ethanol; RX-20001; Resolvyx Pharmaceuticals, Bedford, MA) was injected i.p. (200 µl; 114 µg/kg +1.4% ethanol in saline) 1 h before and 1, 3, 5, and 7 days after laser treatment. Control mice were treated similarly but only received 1.4% ethanol in saline (vehicle). Mice were anesthetized and their pupils dilated for laser treatment.

### Laser choroidal neovascularization

Laser treatment was performed on each mouse retina. A coverslip, floated on a drop of 2.5% methylcellulose on the mouse cornea, served to subtract the optics of the cornea and lens for optimal viewing of the retina and placement of the laser lesions. To trigger the onset of retinal CNV, three 532-nm diode laser spots (lesion diameter 50 µm; duration 100 ms; energy 150 mW) were applied to each fundus with a Novus Spectra ophthalmic laser (Lumenis, Inc., Santa Clara, CA) mounted on a slit lamp (Model SL-07; Topcon, Inc.,Tokyo, Japan). These lesions were placed between retinal vessels two to three disc diameters from the optic nerve in an isosceles triangle with the apex in the superior retina, making it possible to identify each lesion in the flatmount preparations during analysis. Our inclusion criteria were the formation of a bubble immediately after laser application and the absence of subretinal hemorrhage. Lesions violating these criteria were excluded from the study. After full recovery from anesthesia, mice were returned to the animal colony.

### Angiography

In vivo fundus fluorescein angiography of CNV lesions was conducted using a SPECTRALIS^®^ high-resolution, spectral domain optical coherence tomography (HRA+OCT) imaging system (Heidelberg Engineering, Inc., Vista, CA), which uses high-resolution spectral domain optical coherence tomography and a confocal scanning laser ophthalmoscope.

Fluorescein treatment (0.02 ml of 25% fluorescein, Hub Pharmaceuticals, Rancho Cucamonga, CA) delivered once i.p., initiated a two-image leakage analysis protocol, which was performed at post-laser treatment days 7 and 14. After fluorescein injection, early and late-phase fundus angiograms were obtained at an interval of 5 min. The early phase angiogram was obtained 1 min after injection; timing started immediately after fluorescein injection.

### Leakage grading and area analysis

**Figure 1:**
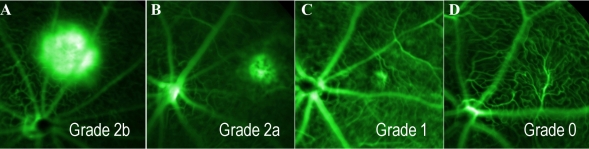
Fundus angiography illustrating the grading scale used for leakage cloud classification. Following an intraperitoneal fluoroscein injection, angiograms were obtained after 1 and 6 min to determine the rate of leakage. Retinal images were collected and graded from control and neuroprotectin D1 (NPD1)-treated animals at 7 and 14 days following laser treatment. The terms Grade 0, Grade 1, Grade 2a, and Grade 2b are defined as follows: Grade 0 (**D**), no hyperfluorescence; Grade 1 (**C**), hyperfluorescence without leakage; Grade 2a (**B**), hyperfluorescence and late leakage; Grade 2b (**A**), bright hyperfluorescence and late leakage beyond treated areas.

The leakage of each CNV lesion was graded by a retina specialist according to the method described by Marneros et al. [28]. Briefly, early and late-phase angiograms were compared to determine whether the lesion exhibited bright hyperfluorescence and late leakage beyond treated areas (Grade 2b); hyperfluorescence and late leakage (Grade 2a); hyperfluorescence without leakage (Grade 1); or no hyperfluorescence (Grade 0; Figure 1). Only lesions with a leakage of Grade 2b were considered clinically significant. Additionally, the leakage area for each lesion was measured using a custom programmed macro for National Institutes of Health (NIH) ImageJ software. Early and late-phase angiograms of each lesion were presented serially to an observer who outlined each fluorescent cloud, using a Wacom drawing tablet interface (Wacom Technologies, Corp., Vancouver, WA); treatment groups and recovery durations were randomized and blinded from the observer. The software recorded treatment, angiogram phase, and leakage area values.

### Immunofluorescence and volumetric analysis

At 7 and 14 days after laser application, mice were sacrificed by cervical dislocation, eyes were enucleated, and corneas slit. Anterior segments and retinas were removed to create an eye cup following overnight fixation (4 °C) in 4% paraformaldehyde (Electron Microscopy Sciences, Hatfield, PA) in phosphate buffered saline (PBS, pH 7.4; Grand Island Biologic Company, St. Louis, MO). Eye cups were washed in PBS (3×10 min), permeabilized, and then blocked with 2% normal donkey serum (Sigma, St. Louis, MO) and 1% Triton-X (Invitrogen) in PBS (3×10 min). Eye cups were then incubated (overnight, 4 °C) with Isolectin B_4_, *Griffonia simplicifolia*, conjugated to AlexaFluor^®^ 568 (25 µg/ml; Invitrogen), phalloidin conjugated to AlexaFluor^®^ 488 (5 units/ml; Invitrogen), and Hoechst 33258 (20 µg/ml; Invitrogen) to detect endothelial cells, cytoskeleton (f-actin), and nuclei, respectively. Following a final wash with PBS, labeled eye cups were flattened with four peripheral radial cuts and coverslipped with ProLong Gold antifade medium (Invitrogen). Flatmounts were imaged on a Zeiss LSM-510 Meta laser confocal microscope with a 40× oil-immersion objective (Zeiss Plan-NEOFLUAR 40×/1.3 Oil DIC; Zeiss, Thornwood, NY). Optical slice thickness for all fluorophores was 0.9 µm. Image resolution was set to 0.45 µm per pixel. Cubic voxel dimensions were ensured by setting the z-step interval to 0.45 µm. Visualization of the fluorescent signal was achieved as follows (excitation; emission; pinhole Ø): Hoechst 33258 (405 nm; 420–490 nm; 1.27 Airy); AlexaFluor^®^ 488 (488 nm; 505–550 nm; 1.00 Airy); AlexaFluor^®^ 568 (543 nm; 560–615 nm; 0.94 Airy). For each lesion, an image stack was collected from the RPE surface through the choroid; the upper and lower boundaries in the z-direction were adjusted to include all possible isolectin signals. Volumetric analysis of these three-dimensional (3D) images was done using a custom programmed plug-in for NIH ImageJ software. The image stack histogram of the target channel (isolectin) was normalized, ensuring inclusion of low intensity signals. Noise generated by normalization was smoothed using a modified 3D median filter operation, which better preserved the true 3D signal over the noise. After median filtering the target channel was segmented with an adaptive 3D thresholding algorithm and binarized. Inclusion of a pixel was calculated based on continuity comparison to pixels contained within a user-defined 3D radius. The volume was then calculated by summing the number of included pixels and multiplying by the x, y, and z dimensions of the voxel unit.

### Statistics

All statistical analyses of clinical grading and leakage area were done using the statistical analysis software, SAS v9.1 (SAS Institute Inc., Cary, NC). For clinical grading, three CNV lesions per eye were induced by laser in 70 mice (40 controls; 30 NPD1). Of the 420 lesions, only one control lesion was rejected due to hematoma (see Laser choroidal neovascularization section). Cataracts developed in seven eyes (six control; one NPD1), preventing angiographic analysis. Before the day 7 angiograms, two control-treated mice died and one additional control mouse died before the day 14 angiography. All lesions within an animal were tabulated by grade category and distinct treatment–recovery interactions to create a frequency table with 540 observations: control-7d (38 mice, 209 lesions), NPD1–7d (30 mice, 177 lesions), control-14d (37 mice, 203 lesions), NPD1–14d (30 mice, 177 lesions). Distributions of clinical grades were fit to the generalized logit model by logistic regression, and individual treatments were contrasted using the asymptotic chi-square distribution of the Wald statistic (n=766, α=0.05). Leakage area data were fit to a general linear model by the least-squares method and compared by Analysis of Variance (ANOVA): control-7d n=47, NPD1–7d n=56, control-14d n=47, NPD1–14d n=56, α=0.05. Only lesions having angiograms at both 7 days and 14 days were included, and the late-phase leakage was modeled as a function of treatment, recovery duration, and early phase leakage. Least-squares-adjusted means and standard error were calculated from the model and used for data representation. CNV-complex volumes were compared by one-way ANOVA using Microsoft Excel 2007 (Redmond, WA): control-7d n=7, NPD1–7d n=7, control-14d n=7, NPD1–14d n=7, α=0.05.

## Results

### Neuroprotectin D1 reduces fluorescein leakage of laser-induced choroidal neovascularization lesions

**Figure 2:**
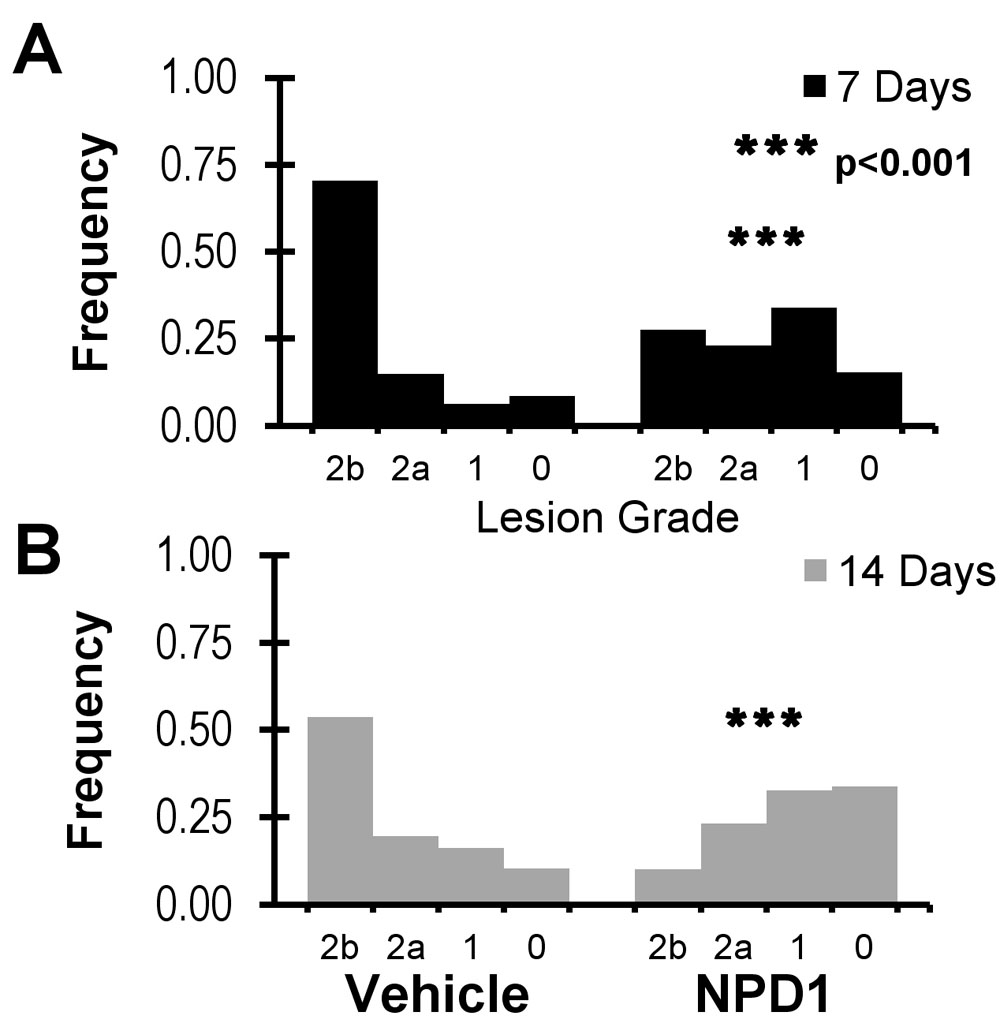
Analysis of leakage clouds from retinas of control and neuroprotectin D1 (NPD1)-treated animals at 7 and 14 days following laser treatment. **A**: Comparison of the frequency of occurrence of leakage grades 7 days after laser damage in control and NPD1-treated retinas, illustrating a significant shift in NPD1 retinas away from the Grade 2b lesions of controls into grades of lesser damage. **B**: A similar comparison, 14 days following laser treatment, showing a continual shift from Grade 2b lesions toward Grades 0, 1, and 2a and a reduction of leakage from the NPD1 treatment. Asterisks denote level of statistical significance as compared to vehicle (*** p<0.001).

CNV leakage in NPD1-treated and vehicle-treated mice was assessed by fluorescein angiography [28]. The distribution of lesion grades was plotted for each treatment at 7 and 14 days post laser (Figure 2). In vehicle-treated mice, the duration of recovery following laser CNV initiation did not affect leakage severity. Treatment with NPD1, however, resulted in a difference in leakage grade distribution. The leakage distribution at day 14 was skewed toward a lower severity when compared with the day 7 distribution (Figure 2A,B). CNV leakage at 7 days demonstrated remarkable differences in grade distributions between treatments. Mice treated with the vehicle had 43% more severe Grade 2b leakage than NPD1-treated mice, while less severe Grade 1 leakage in NPD1-treated mice was over fivefold higher than in vehicle-treated mice (Figure 2A). This trend continued at 14 days of recovery, with NPD1-treated lesions exhibiting twofold and threefold higher levels of Grade 1 and Grade 0 leakage, respectively, and vehicle-treated lesions having fivefold higher Grade 2b leakage (Figure 2B).

**Figure 3:**
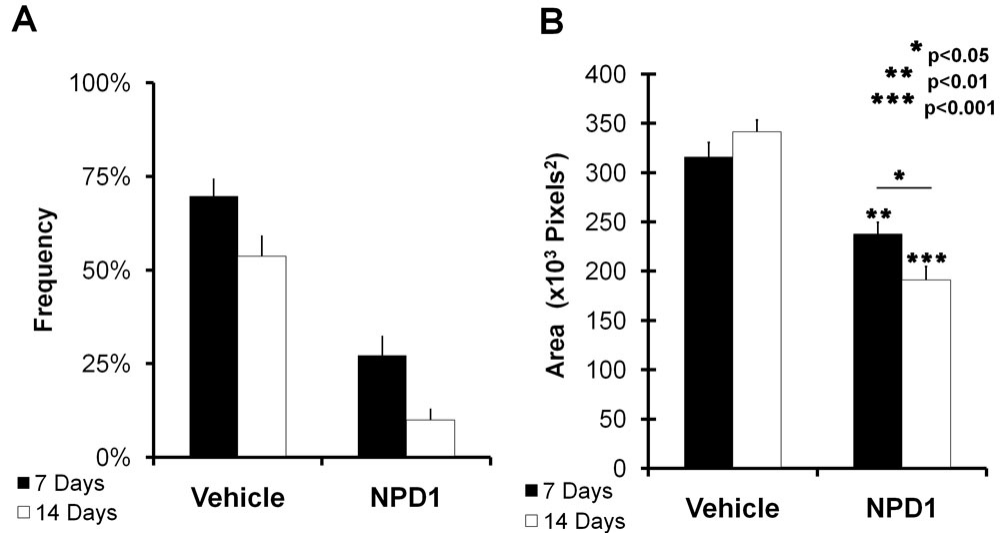
Angiographic analysis of choroidal neovascularization (CNV)-associated leakage clouds. **A**: The graph represents the frequency of occurrence of clinically relevant lesions. The percent occurrence of only lesions considered clinically analogous to human lesions are shown. This comparison of lesions from control and neuroprotecin D1 (NPD1)-treated animals at 7 and 14 days post-laser treatment (from Figure 2) shows an approximate reduction in occurrence of >40% when NPD1 retinas are compared to controls. **B**: The graph represents the leakage cloud areas from all laser lesions. Following administration of fluorescein (intraperitoneal), the resulting cloud formed at each lesion site was imaged at 1 and 5 min. Early and late images of each lesion were presented serially in a randomized and blind fashion to an observer who manually outlined the leakage area. The graph represents adjusted means produced using a general linear model. Leakage growth was significantly lower in NPD1-treated animals than in controls at both 7 and 14 days. Asterisks denote level of statistical significance as compared to vehicle (*p<0.05; **p<0.01; *** p<0.001).

Treatment comparison of clinically relevant leakage revealed that NPD1-treated mice had 60% and 80% fewer relevant lesions than vehicle-treated mice at 7 and 14 days recovery, respectively (Figure 3A). Additionally, both treatment groups demonstrated a decrease in clinically relevant leakage as recovery duration increased. While the group treated with vehicle exhibited a 24% further reduction in relevant lesions between days 7 and 14 after laser initiation of CNV, the NPD1-treated animals had a much larger reduction (63%).

The fluorescein leakage of each lesion was measured in early and late angiograms, and the leakage area was compared using a general linear model (see Methods) at 7- and 14-day recovery durations. Comparisons of lesions in NPD1-treated animals with those of control mice are plotted in Figure 3B. After 7 days of recovery, the leakage area had been reduced by 25% in the NPD1 group, and by 14 days NPD1 reduced leakage by 44%. Unlike the grade distributions, increased recovery duration alone did not reduce leakage, as seen by the comparison of 7- and 14-day leakages in vehicle-treated mice. With NPD1 treatment, however, increased recovery reduced 7-day leakage by 19%.

### Endothelial cell proliferation at lesion sites is reduced with neuroprotectin D1 treatment

**Figure 4:**
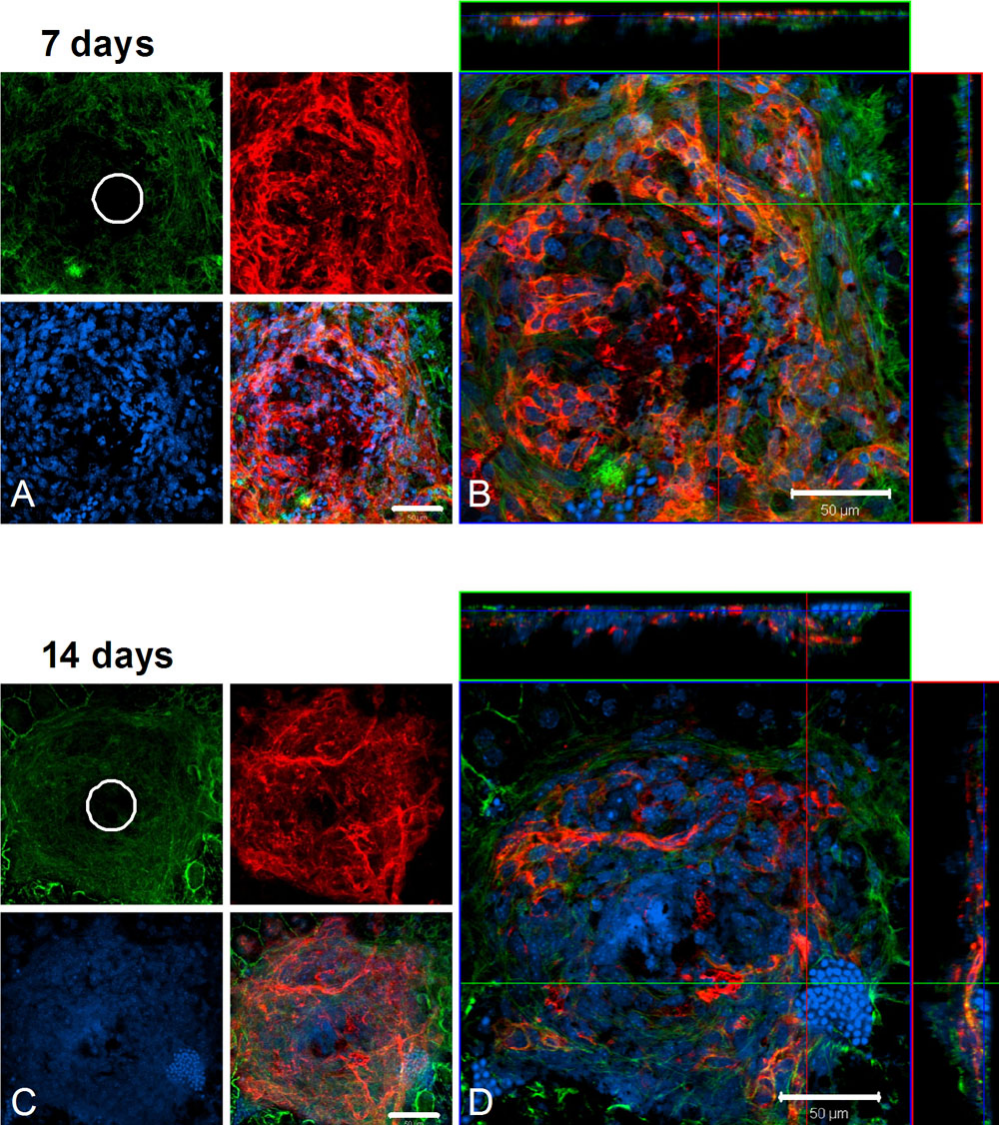
Confocal microscopy of laser-induced choroidal neovascularization (CNV), showing extent of endothelial cell proliferation in control animals. **A, B**: In this flatmount preparation of a control choroid at 7 days post treatment, the retina has been removed to expose a typical laser lesion through Bruch’s membrane and into the choriocapillaris. **A**: Maximum projection (brightest voxel displayed for each Z-column) images of immunolocalization for cytoskeleton (f-actin, green), nuclei (Heochst, blue), and endothelial cells (isolectin B4, red) are shown on the left, along with a merged image of the three labels. The initial lesion (50 µm diameter) is apparent in the f-actin image, while endothelial cells (capillaries) are seen to fill the site as proliferation proceeds. **B**: The same lesion as in **A**, presented as an orthogonal cut view. The large square image represents a single layer in a confocal stack of images, as viewed above from the retina, with the three labels merged. Lateral views of this image stack are shown at the top and to the right; the blue line in these views indicates the level within the stack of the view shown in the square. The lateral view at the top is from the position of the green line in the large square; the lateral view to the right is from the position of the red line in the large square. **C, D**: Images are organized the same as in **A** and **B** and depict a typical laser lesion of a control retina at 14 days post treatment. White circles represent the 50-µm-diameter initial laser lesion. Magnification bars equal 50 µm.

Figure 4A illustrates maximum projection views of a typical 7-day control lesion in which the green cytoskeleton (f-actin), blue nuclei, and red endothelial cell channels are separated. The site of the laser lesion is apparent in the green cytoskeleton channel, and extensive endothelial cell ramification is evident in the red channel. The merged image represents the superimposition of these three channels, illustrating one layer within the confocal image stack. While the initial laser lesion was 50 µm, the lateral extent of endothelial cells outward from the lesion at 7 days approached 200 µm. Neovascularization occurred near the upper surface in the lateral views of the stack, which corresponds to the RPE cell layer. At 14 days after laser injury, labeled control choroidal flatmounts retained their extensive endothelial cell lateral distribution and outward distribution toward the retina (Figure 4B).

**Figure 5:**
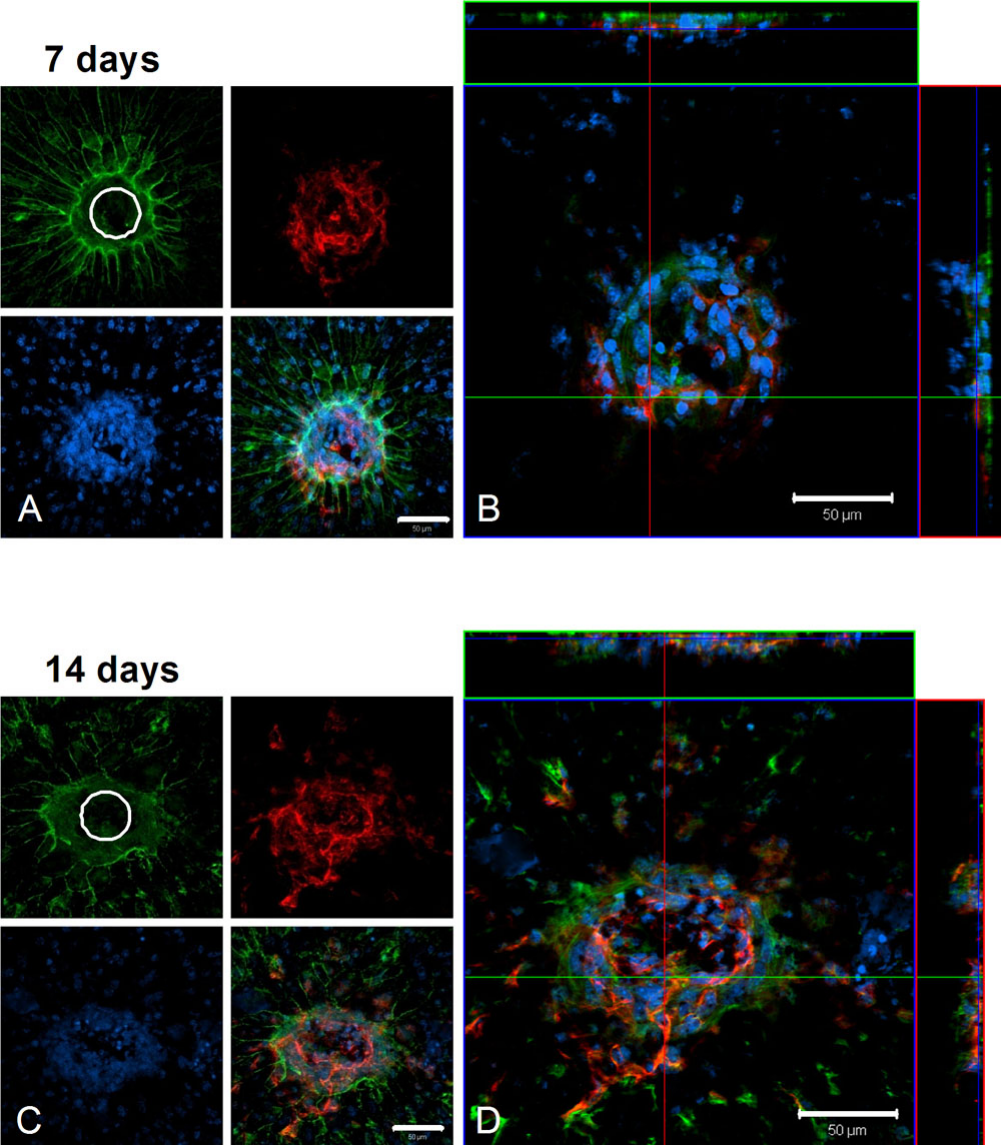
Confocal microscopy of laser-induced choroidal neovascularization (CNV) shows the extent of endothelial cell proliferation in neuroprotecin D1 (NPD1)-treated animals. **A, B**: In this choroidal flatmount preparation from an NPD1-treated animal at 7 days post treatment, the retina has been removed to expose a typical laser lesion through Bruch’s membrane and into the choriocapillaris. **A**: Maximum projection images of immunolocalization for cytoskeleton (f-actin, green), nuclei (Heochst, blue), and endothelial cells (isolectin B4, red) are shown on the left, along with a merged image of the three labels, as in Figure 4. **B**: The same lesion as in **A**, presented as an orthogonal cut view. The large square image represents a single layer in a confocal stack of images, as viewed above from the retina, with the three labels merged. Lateral views of this image stack are shown at the top and to the right; the blue line in these views indicates the level within the stack of the view shown in the square. The lateral view at the top is from the position of the green line in the large square; the lateral view to the right is from the position of the red line in the large square. **C, D**: Images, organized the same as in **A** and **B**, through a typical retinal laser lesion of an NPD1-treated animal at 14 days post treatment. This lesion is presented at the right as an orthogonal cut view. White circles represent the 50-µm diameter initial laser lesion. Magnification bars are 50 µm.

The effect of NPD1 treatment on endothelial cell proliferation is evident from choroidal flatmounts. Confocal imaging, as with the control groups, revealed cytoskeleton, nuclei, and endothelial cell distributions. At 7 days after laser treatment, the initial lesion was visible in the green cytoskeleton channel, but some overcapping of the RPE cells (outlined in green) was apparent. Again, the endothelial cells ramified within the lesion, but their lateral distribution remained at about 50 µm, the diameter of the laser beam (Figure 5A). There was an increase in the endothelial cell lateral array to about 100 µm after 14 days (Figure 5B), but the distribution of cells in the confocal stacks associated with these lesions remained at one-third to one-half the size of those found in the control groups at both 7 and 14 days.

**Figure 6:**
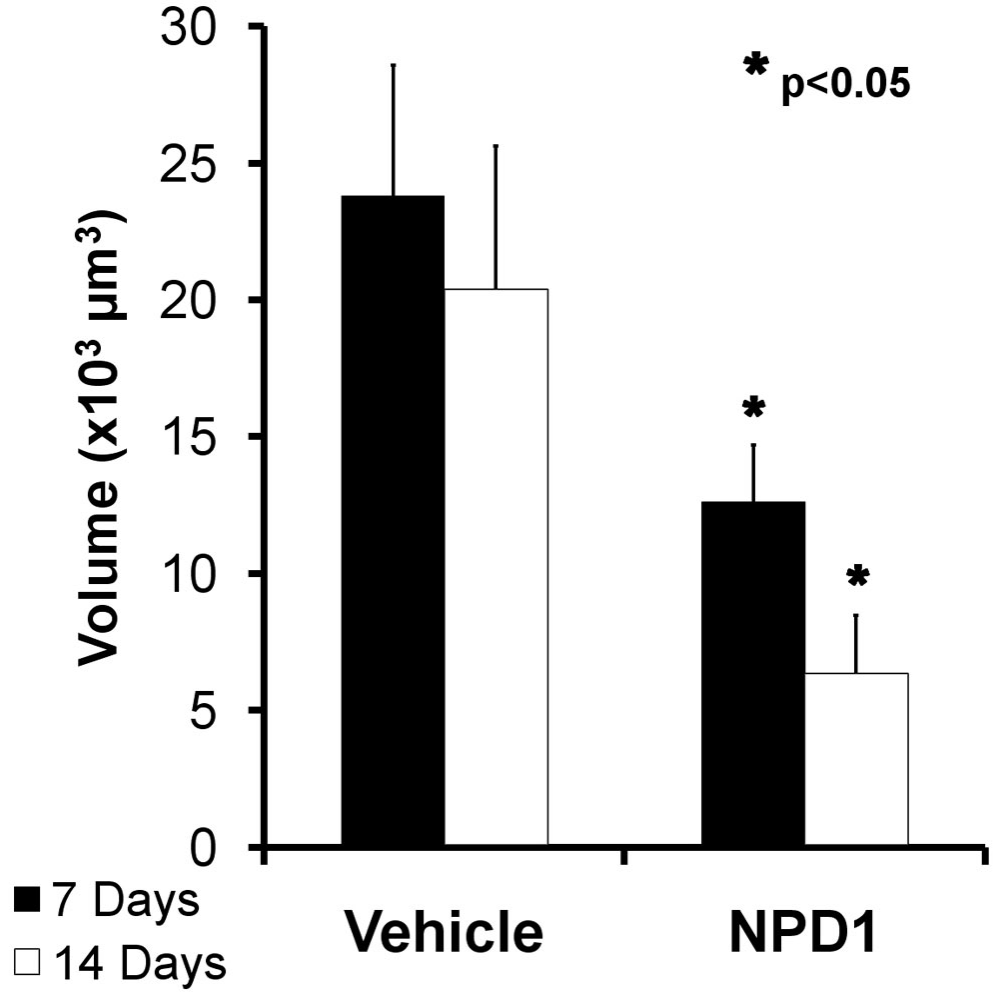
Comparison of endothelial cell volumes within laser-induced lesion sites in choroids of control and neuroprotecin D1 (NPD1)-treated mice. Image stacks were obtained through each laser lesion by confocal microscopy, and all voxels from the isolectin B4-labeled endothelial cell channel with intensities above threshold were summed for each layer. All layers throughout the stack were then summed to give the total number of endothelial cell voxels per lesion at 7 and 14 days following laser treatment. Volumes were calculated by multiplying the total number of voxels by the voxel dimension (0.45 µm×0.45 µm×0.45 µm). These volumes are represented by the black (control) and white (NPD1-treated) bars. Asterisks denote level of statistical significance as compared to vehicle (*p<0.05).

Finally, volumetric analysis of isolectin-B_4_ revealed endothelial cell volumes within each lesion at days 7 and 14. Control lesions contained about 24,000 µm^3^ of endothelial cell labels at day 7, compared to NPD1 lesions with about 13,000 µm^3^, which represents about 46% less neovascularization. The effect was more dramatic at 14 days with about 20,000 µm^3^ of endothelial cell labels in control groups and about 6,500 µm^3^ in the NPD1-treated group. This represents a reduction in neovascularization volume at 7 and 14 days post-laser damage by about 50% and 66%, respectively (Figure 6).

## Discussion

In the present study, we found that NPD1 significantly reduced the extent of clinically relevant vascular leakage based on an established clinical grading scale. Importantly, NPD1 was administered only during the first week of recovery, yet observations of NPD1-treated mice at day 14 demonstrated continual reduction of leakage even after cessation of treatment. The effect of NPD1 was also observed as a reduction in leakage rate, indicating that NPD1 had a direct effect on the underlying dynamic pathogenic process throughout the observation period. Ramification of endothelial cells displayed a similar response to NPD1 treatment. Endothelial cell volume was significantly lower at 7 days of recovery time, and as with leakage, reduced endothelial cell volume was not simply maintained after cessation of NPD1 treatment but continued to decrease with further recovery time. In contrast, vehicle treatment demonstrated only a modest reduction in clinical grading by the end of the second week but failed to reduce leakage or endothelial cell growth, indicating that the enduring inhibition elicited by NPD1 was not a simple component of normal resolution in the CNV model.

It is possible that the reduction of laser CNV leakage by NPD1 occurs through inhibition of NF-κB activation and COX-2 expression [27]. Retinal choroidal cells stimulated with IL-1β resulted in increased COX-2 and ultimately VEGF expression [4]. Moreover, we have shown that NPD1 downregulates IL-1β and TNF-α gene expression [29], which are known to increase permeability of the pericyte/endothelial cell complex [30]. Thus, NPD1 inhibition of CNV-associated leakage may be the combinatorial result of reducing IL-1β and TNF-α expression and inhibiting the activity of NF-κB, a common downstream transcription factor of both pathways. This would result in decreased permeability (vascular leakage) at the pericyte/endothelial cell complex and a decrease in VEGF expression, which would reduce growth and permeability of endothelial cells.

A second mechanism that could be involved in limiting pathoangiogenesis in our laser CNV model is promotion of cell survival. RPE cell integrity/viability is a key component in the pathology of both dry and wet versions of AMD. Promoting RPE cell survival could play a role in re-establishing Bruch’s membrane, reforming of the RPE junctional complex, and isolation of the subretinal space, thereby preventing inappropriate molecular communication between the choroid and subretinal space. We have shown that NPD1 potently protects the RPE from oxidative stress/inflammatory-induced apoptosis [23,24]. NPD1 inhibits inflammatory beta-amyloid-42 (Aβ-42)-induced apoptosis of human neural cells composed of both neurons and glia [29], and retinal ganglion cell apoptosis, triggered by optic nerve damage, is prevented [31], suggesting that NPD1 may also protect photoreceptors and Müller cells from CNV inflammatory signaling. The undamaged periphery of the initial laser lesion, or penumbra, may be subject to secondary degeneration that results in a slow expansion of CNV. While the RPE, Müller cells, and photoreceptors in this penumbra are not obliterated by the laser damage, they may be severely stressed, leading to enlargement of the lesion. Studies in a mouse CNV model illustrate that neutropenia prevents enlargement of retinal laser lesions, while lesions in wild-type mice double in size, showing that neutrophils (polymorphonuclear leukocytes-PMNs) promote the expansion of CNV [32]. In stroke, a key reason for the expansion of the penumbral region is infiltration of PMNs and ameboid microglia [33]. Moreover, NPD1 prevented PMN infiltration and markedly reduced penumbral damage in an ischemia-reperfusion model [27]. This suggests that NPD1 may rescue cells in the penumbral region of laser-induced CNV by directly promoting RPE survival and reducing PMN infiltration. This may result in containment of the lesion and preservation of the penumbral glia and neurons.

We have shown that NPD1 inhibits choroidal neovascularization. The efficacy in this model may be a function of the combinatorial actions of NPD1 in protecting cells from apoptosis while downregulating the pro-inflammatory cytokines TNF-α and IL-1β, possibly by inhibiting NF-κB activity and reducing COX-2, leading to decreased VEGF expression. Overall NPD1 may contribute to fine tuning the induction of the resolution phase of the inflammatory response. The anti-angiogenic and neuroprotective actions of NPD1 highlight its potential applicability in treating wet AMD and diabetic retinopathy, and the current results show NPD1 to be at least as efficacious as, if not more than, other interventions explored in this model. Based on our understanding of the pathology of the CNV model, the results imply the potential suitability of NPD1 for the treatment of other neural (e.g., Alzheimer disease) and non-neural (e.g., asthma) inflammatory diseases.
